# Hospital-Based Healthcare Workers Victims of Workplace Violence in Italy: A Scoping Review

**DOI:** 10.3390/ijerph18115860

**Published:** 2021-05-29

**Authors:** Cristina Civilotti, Sabrina Berlanda, Laura Iozzino

**Affiliations:** 1 Department of Psychology, Università di Torino, Via Verdi 10, 10124 Torino, Italy; cristina.civilotti@unito.it; 2 Department of Human Sciences, University of Verona, Lungadige Porta Vittoria, 17, 37129 Verona, Italy; sabrina.berlanda@univr.it; 3 Psychiatric Epidemiology and Evaluation Unit, IRCCS Istituto Centro San Giovanni di Dio Fatebenefratelli, Brescia, Via Pilastroni 4, 25125 Brescia, Italy

**Keywords:** workplace violence, hospital, healthcare workers, nurses, physicians, Italy

## Abstract

The aim of this scoping review is to synthesize the available evidence on the prevalence rates of healthcare workers being victims of violence perpetrated by patients and visitors in Italy. PubMed, Scopus, Web of Science and CINAHL were systematically searched from their inception to April 2021. Two authors independently assessed 1182 studies. All the scientific papers written in English or in Italian reporting primary quantitative and/or qualitative data on the prevalence of aggression or sexual harassment perpetrated by patients or visitors toward healthcare workers in Italy were included. Thirty-two papers were included in the review. The data extracted were summarized in a narrative synthesis organized in the following six thematic domains: (1). Methodology and study design; (2). Description of violent behavior; (3). Characteristics of health care staff involved in workplace violence (WPV); (4). Prevalence and form of WPV; (5). Context of WPV; and (6). Characteristics of violent patients and their relatives and/or visitors. The proportion of studies on WPV differed greatly across Italian regions, wards and professional roles of the healthcare workers. In general, the prevalence of WPV against healthcare workers in Italy is high, especially in psychiatric and emergency departments and among nurses and physicians, but further studies are needed in order to gather systematic evidence of this phenomenon. In Italy, and worldwide, there is an urgent need for governments, policy-makers and health institutions to prevent, monitor and manage WPV towards healthcare professionals.

## 1. Introduction

Compared to other forms of violence, the interest shown in workplace violence (WPV) has grown over the years [1,2]. In particular, violence against healthcare workers perpetrated by patients or visitors presently has the traits of an emergency. Paradoxically, taking care of suffering people may become a risky duty; hospitals may become violence-prone workplaces, and health workers are often “assaulted and unheard” [3,4]. WPV threatens, in fact, the well-being of both the workers and the organization they belong to, negatively influencing the rights of millions of people to work in a safe environment and affecting the organization with absenteeism and low productivity, among other things. For these reasons, worldwide, several international organizations [5,6,7], along with many different research groups [8,9,10,11,12], are underlining the importance of having specific guidelines to monitor and prevent the spreading of this phenomenon. In the last two decades, the number of scientific publications on this topic has grown. Literature is organized into primary studies and meta-analytical reviews. In primary studies, data are collected in order to describe the phenomenon of violence in a specific context (e.g., emergency or psychiatric departments) [13,14], toward a target population (e.g., oral health-care workers) [15], in a particular geographical area (e.g., African or European Countries) [16,17] or in specific countries (such as China, Spain or Iran) [18,19,20]. Meta-analytical reviews e.g., [21,22], allowing a general perspective in the evaluation of the phenomenon, by merging and/or re-reading the data or the conclusions drawn from previous works. This paper pertains to the second category, consisting of a scoping review aimed at describing the prevalence of healthcare staff (physicians and nurses and technicians) who have been subject to violence or aggression by patients or visitors in Italy.

### 1.1. Critical Issues on Definitions and Complexity Elements in Studying the Concept of Violence and Workplace Violence

A first element of complexity in studying WPV pertains to the definition of “violence” itself. To overcome this problem, the solution accepted by most scholars is to use a definition of violence that is generalized and generalizable, since the components and declination of violence are multiple and variegate [23]. For the aim of this paper, we refer to the World Health Organization [24], which describes violence as, “The intentional use of power, threatened or actual, against another person or against a group, in work-related circumstances, that either results in or has a high degree of likelihood of resulting in injury, death, psychological harm, maldevelopment, or deprivation”. Two fundamental aspects emerge from this definition. First of all, violence is considered an intentional act, committed by an agent who actively wants to harm another person. The second key point is that the idea of violence as a mere physical injury has been replaced by a broader concept that includes also psychological harm [25].

Although the concept of WPV is more specific than the concept of violence, for it encompasses a narrower field, there is not a unique definition in the literature. It is described using a wide range of words and situations that differ from each other, from physical and sexual aggression to verbal, mental and/or moral abuse [25,26,27,28,29,30,31]. A further complexity in identifying and classifying WPV is related to the contextual influences, as pinpointed by Waddington and colleagues [23]. The concept of violence cannot be detached from the socio-cultural context in which it is embedded. As stated by Escribano and colleagues [3], “workplace aggression does not occur in a social vacuum”, but it emerges in an interconnected framework that includes both organizational and psychosocial factors [32,33,34].

Since the socio-cultural context may play a significant role in studying the WPV phenomenon, as already done previously in other studies, e.g., [19,20], the aim of the present scoping review is to give a comprehensive picture of it in a European country, namely Italy.

### 1.2. Legal Aspects of WPV in Italy

For a long time in Italy, there has not been a specific law regarding violence in the workplace, nor any law concerning violence specifically designed for the health sector. The only existing protocol to control violent behavior in the health area was the one developed in 2005, initially on an experimental basis, by the Ministry of Labor, Health and Social Policies, named Protocol for Sentinel Events Monitoring. The latest version of this document dates back to 2009. It is a ministerial circular, a regulatory act that encourages and promotes good practices through the analysis of violent behavior. Its purpose, in fact, was to observe and monitor the so-called “sentinel events”, with the aim of defining this type of events in the same way at a national level. The objective of the Protocol for Sentinel Events was to have the same vision and the same line of action between Regions, Provinces and local health authorities operating in the area, to guarantee an Essential Level of Assistance. In recent years, to evaluate WPV in health sectors and contain its effects, various guidelines, good practices and recommendations have been issued by trade associations, the Ministry of Health, etc. However, these tools did not favor a systemic and quantitative approach to risk assessment or to identifying in an organic way the prevention and protection measures for the health care workers. Only very recently, with the law no. 4 (15 January 2021), the first in Europe, the Italian President of the Republic ratified the Convention of the International Labor Organization (n. 190) on the elimination of violence and harassment in the workplace, initially adopted in Geneva on 21 June 2019. The Convention may become an important tool in combating violence and sexual harassment in the workplace. First, as we did in this study, it provides the broad, internationally approved definition of violence of the World Health Organization (harassment or violence is any behavior likely to cause physical, psychological and economic harm). Furthermore, explicit reference is made to discrimination based on gender, recognizing that women are particularly exposed to physical, economic and psychological aggression in the workplace.

The aim of this work is to deepen the knowledge of violence in the workplace in healthcare settings within the Italian context, in order to lay the foundations for the structuring and the implementation of future prevention and containment interventions. For the purpose of this paper, we adopted the widely used classification in four main categories proposed by the University of Iowa Injury Prevention Research Center [35] (Westlawn, Iowa, with a specific focus on Type II workplace violence (in Type II, a customer, a patient, a client or an inmate is responsible for it, and the staff is the victim).

## 2. Materials and Methods

This scoping review was conducted following the procedures indicated by Arksey and O’Malley [36] and the subsequent amendments proposed by Levac and colleagues [37] and Daudt and colleagues [38]. The scoping review is a technique used in order to synthesize a segment of scientific knowledge related to a specific field, frequently used when (i) it is difficult to accurately identify a narrow review question (as with the phenomenon of workplace violence, in the context of this paper); (ii) the studies used as sources are likely to have used a wide range of data collection and analysis methods; (iii) no previous meta-analyses or systematic reviews have been made relating to the topic; and (iv) the quality assessment of the selected studies is not going to be conducted. Having met three of the four conditions (i, iii and iv), this scoping review has the primary objective of investigating the prevalence of health workers as victims of violence and describing the main characteristics of the phenomenon using the available data relating to the Italian context.

### 2.1. The Scoping Review Question

The first step was the definition of the scoping review question, which was postulated starting from the analysis of the national and international scientific literature regarding the phenomenon of violence in the workplace in the hospital context and the relevant Italian legislative system. The research question was specifically aimed to collect information about the prevalence of healthcare staff (physicians and nurses and technicians), working in public or private hospitals, who have been subject to violence or aggression by patients or visitors. For these reasons, behaviors such as bullying or mobbing were excluded because they usually are perpetrated by colleagues or employers.

### 2.2. Search Strategy

The research keys were chosen with the aim of maximum inclusiveness, starting with the review of relevant literature and ultimately through team consensus. The search strategy used was structured as follows: (hospital * OR healthcare OR health OR nurse * OR doctor * OR physician * OR surgeon * OR psychiatry * OR obstetric *) AND (violence OR aggression * OR harassment) AND Ital *.

Four electronic, databases, Scopus, WoS (Web of Science) and CINAHL were used. The search was conducted up to 28 April 2021 on article title, abstract and keywords fields, without any restrictions of language or time interval. All the studies corresponding to these inclusion criteria were included:

- Original research papers and scientific reports;
- Publications written in English or Italian (languages spoken by the authors);
- Articles reporting primary quantitative and/or qualitative data on the prevalence of healthcare workers as victims of aggression or sexual harassment perpetrated by patients or visitors.

Review and meta-analytical papers, general comments or discussions in scientific journals or in opinion papers, editorials, case reports and book chapters were excluded.

### 2.3. Screening Procedure

Articles were selected by title and abstract by two independent reviewers, CC and SB (who both have at least 10 years of academic experience in, respectively, clinical and social psychology). Full texts considered as eligible by at least one of the two reviewers were included in the preliminary selection. In case of discord about the inclusion of a full text, a third reviewer (LI, with 10 years of experience in psychology in the public health context) was consulted, in order to reach an agreement.

### 2.4. Data Extraction and Results Reporting

For each study included, the following variables were extracted: prevalence of healthcare workers as victims of aggression or sexual harassment perpetrated by patients or visitors, publication year, geographical collocation, kind of sample, response rate and tool(s) used. Data extracted were summarized in a narrative synthesis organized in the following six thematic domains: 1. Methodology and Study design, 2. Description of violent behaviors, 3. Characteristics of health care staff involved in WPV, 4. Prevalence and form of WPV, 5. Context of WPV and 6. Characteristics of violent patients and their relatives and/or visitors.

## 3. Results

**Figure 1:**
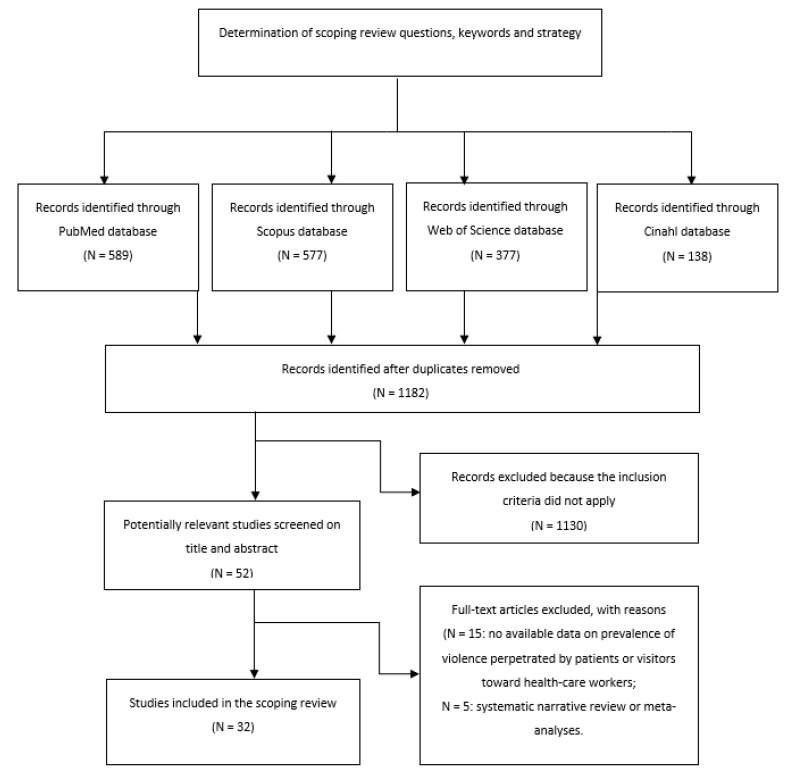
Flow chart of the search strategy and results.

Overall, after removing duplicates, the search has reported 1182 records. After screening for title and abstract, we removed 1130 papers and we assessed the eligibility of 52 full-texts. Twenty articles were then excluded for different reasons, such as mismatch with inclusion criteria and unavailability of the full text (see Figure 1). A total number of 32 articles was included in the systematic narrative review.

### 3.1. Methodology and Study Design

**Figure 2:**
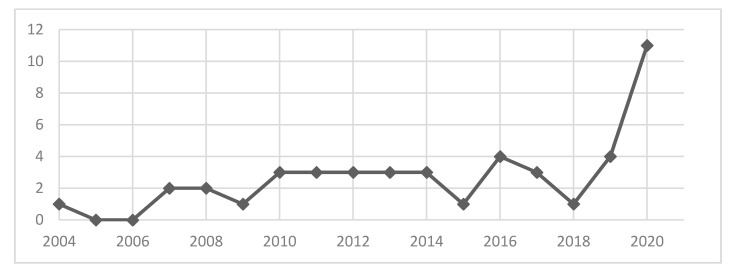
Number of included publications per year from 2004 to 2020. 2021 was excluded because is still ongoing.

The list of included studies is shown in Table 1. All the studies selected were retrospective cross-sectional surveys that focused on staff experiences with different forms of WPV in different clinical contexts. Even without any restrictions of time, we found studies only starting from 2004. In the course of the investigated period (up to 2021), a steady increase from less than one study per year (2004–2010) to more than three studies per year (2010–2021), with a peak of publications in 2020 (see Figure 2), was observed. This was considered indicative of a growing interest in the subject over the past decade.

Before 2004, there was an interesting paper [39] that presents a violence-prevention plan developed in a psychiatric unit and reports its effects on assault rates; in it, the assault rates from 1995 to 2009 are presented.

Of the 32 selected studies (Table 1), nine investigated only nurses’ violence experiences (above all in from medical and emergency wards), three looked at medical doctors’ experiences (from medical and psychiatric wards), five focused both on nurses and physician’s violence experiences and all the remaining studies (N = 15) examined the exposition to violence of all the healthcare workers working in general hospitals. Twelve studies in our selection had a sample size of over 500 subjects: the response rate ranged from 7% (in the case of a large national study including all the Italian Emergency Departments) [40] to 96.5% (in the case of a study set in a Local Health Unit in Lazio) [41].

Ten studies investigated the phenomenon based on the occurrence of violent incidents. The most common method used to collect information about the occurrence of violent episodes was the Italian version of the Violent Incident Form-VIF [42], a validated questionnaire for the registration of violent incidents in the healthcare workplace. In five studies, healthcare workers were interviewed about violence experiences using ad hoc questionnaires or semi-structured interviews. Only ten publications provided information on the validity or reliability of the instruments used (those studies in which the VIF was administered). In almost half of the studies, a retrospective time frame of one year was selected (see Table 1). In other four studies, authors investigated the lifetime prevalence of healthcare workers experiencing violence at work. In five studies, the observational period was not clear, and in the remaining ones, the prevalence timeframe was limited to a few weeks up to a few months [14,43,44,45,46,47,48].

### 3.2. Description of Violent Behavior

Of the 32 studies included, seven provided either a minimal definition of violence or none at all [14,32,49,50,51,52,53]. The other publications employed various terms to describe violence or aggressive behavior. These descriptions can be categorized as verbal or physical violence [41,49,53,54,55,56,57,58,59,60,61,62]. In five papers, the definitions used distinguish between verbal aggression (shouting, offenses and threats), physical assault and harassment by describing each behavior in detail [50,63,64,65,66].

### 3.3. Prevalence and Form of WPV

In fifteen studies, participants were asked if they had violent experiences in the last 12 months (Table 2). All the investigated staff groups had experienced more verbal than physical workplace violence. The percentage of healthcare workers experiencing verbal violence ranged from 11.9% (N = 621) to 93.3% (N = 149). In general, studies with a high prevalence rate are those with small samples [57,58,61]. Compared to the prevalence rate of healthcare workers exposed to verbal aggression, the physical violence prevalence rate in the last year is, fortunately, more restrained (from 0 to 53%). Information about the prevalence of healthcare workers who experienced sexual harassment or simply harassment behavior in the past year was available only in three studies.

Four studies contained data about lifetime prevalence of healthcare workers who experienced WPV at some point during their careers (Table 3). Verbal aggression remained the most common type of violence, even though, compared to the last 12 months prevalence, lifetime physical violence prevalence seems to be higher (range = 27.5–50.3%).

### 3.4. Context and Characteristics of Healthcare Workers Involved in WPV

The highest prevalence of violence was observed in general psychiatric wards and emergency departments [57,58].

Five studies [14,43,50,53,58] focused on episodes of aggression experienced by mental health workers in public and private psychiatric services. Psychiatrists are those at the greatest risk of verbal aggression (range = 20.8–97%), injuries (range = 14.3–65.7%), treatment with dangerous objects (72%), object aggression (97% lifetime, 59% in the preceding 12 months), stalking (10.2–19.3%) and physical aggression (range = 17.8–64.6%) compared to other HCWs such as residents, nurses and other professionals.

Prevalence rates of healthcare workers exposed to violence in emergency departments were investigated in six studies [46,47,51,57,61,67]. All the studies confirmed that the majority of HCWs (87%) experienced verbal or physical aggression or both. Nurses are those at greatest risk of verbal or emotional violence (range = 48.2–100%), both verbal and physical violence (range = 7.4–21.7%), physical violence (71%) and sexual harassment (27.4%). Physicians are reported to have experienced WPV less frequently, but still in high percentages (verbal or emotional aggression: 96.5%; physical violence: 39.1%).

Zoleo and colleagues [51] led a study among a general emergency department, an obstetric–gynecological emergency department and a pediatric emergency department. The experience and perception of physical violent events are different across emergency departments. In particular, physical violence was more frequently experienced by workers in general emergency departments.

Sixteen studies [32,41,44,45,48,49,52,55,56,59,60,62,63,65,66,68] focused on prevalence of violence experienced by all the categories of healthcare workers working in general hospitals or healthcare institutions. Across occupations and professional roles, nurses and physicians were the professional groups most exposed to verbal violence (48%). Nurses reported the highest exposure to violence, followed by physicians and other healthcare professionals. Other healthcare professionals included administrative staff, head nurses, health social care workers, midwives, non-medical support staff, nursing assistants and radiologists. More than half of the nurses (range = 85–49.4%) reported having suffered at least one episode of aggression during the previous 12 months. Nurses experienced verbal violence (15.2%) more frequently than physical violence (6–13.4%), unwanted sexual attention and sexual harassment (range = 10.6–15.3% and stalking (range = 14–18.4%). Physicians also experienced verbal violence (range = 34.2–51.53%) more frequently than physical violence (19–3.9%), and unwanted sexual attention (5.4%).

The mean age of staff reporting having been exposed to violence seemed to be lower than that of staff reporting no exposure to violence and less job experience was also found to be indicative of higher risk rates of WPV [54]. In general, young or inexperienced health care staff are at higher risk for WPV, whereas more years of seniority on these jobs seems to be a protective factor for WPV [54]. Overall, female health care staff were more likely to report having been sexually harassed [53].

### 3.5. Characteristics of Violent Patients and Their Relatives and/or Visitors

The six [52,57,59,61,62,64] studies that distinguish between patient and visitors/patients’ relatives as aggressors revealed that patients are clearly the primary aggressors, especially with respect to physical attacks. Verbal aggression by patients’ relatives or friends and visitors is more frequently observed in emergency wards [45].

Patients between the ages of 30 and 50 [46] were found to be the most aggressive. Additionally, the review showed that perpetrators of violence tend to be male [65]. The patient’s health was also an important factor. Abnormal mental states (for example in severe mental disorders or substance use disorders) or cognitive impairments (for example in patients with dementia or mental disability) may contribute to making a patient more physically violent, whereas verbal aggression is more commonly observed in patients without these clinical characteristics [45].

## 4. Discussion

Already visible in other countries [1,4,9,12,15,16,17,18,19,20,21,27,29], the growing interest in the phenomenon of WPV in hospitals is now seen in Italy as well, as demonstrated by the increasing number of scientific publications in the field (in 2020, the highest ever number of published papers was recorded). That said, it is important to note that, although in recent years the total number of publications has generally increased, this does not necessarily imply a discernible trend. Moreover, globally, and particularly in the social and healthcare sectors, there have been more and more frequent aggressions perpetrated by patients and their relatives against health care workers [29]. The question, then, arises spontaneously and legitimately: has attention increased towards this problem because, indeed, more violent acts have been carried out or, simply, because there has been a greater reporting of these episodes? In the research by Estrada and colleagues [1], the authors focused their attention on the change in interest in violent behavior in the workplace and formulated two hypotheses regarding the increase in the number of reported cases. The first hypothesis refers to a causal attribution of a dispositional type. It is investigated whether it is the actors involved in violent acts who are more sensitive and informed with respect to the issue and therefore tend to report those acts with higher frequency. The second hypothesis suggested is, instead, of a situational type. Therefore, it is analyzed whether the growth of the phenomenon of violent behavior is due to working conditions, which are worsening, increasing the exposure of workers to greater risks, including violent acts. The result shows, however, that the answer is to be found in an interplay of the two hypotheses. Taking into consideration only dispositional factors or only situational factors, the phenomenon is not explained in its entirety. Both hypotheses contribute to giving an explanation of this much-debated phenomenon. WPV in the sanitary context is probably affected by various factors at the individual and organizational level; it can negatively influence healthcare workers’ individual along with family relationships (spillover effect) and can lead to a poorer quality of patient care [9,21]. Overall, the causes of this worsening at a global level may be due to increasing socio-economic difficulties, the growing complexity of the healthcare system and a greater diffusion of feelings of anger, frustration and restlessness [9,21].

In Italy, there was a lack of systematic and comprehensive studies on the phenomenon [69]; in spite of this, we were still able to find a certain number of studies, and we can confirm that the type of violence most experienced by Italian healthcare workers—similarly to their colleagues around the world—consists of mainly verbal aggressions and threats (range = 11.9–93.3%) and less frequently physical violence (range = 27.5–50.3%). Ferri and colleagues [45] supposed that, while verbal violence is mainly carried out by people with a balanced mental state, physical violence is usually perpetrated by aggressors with cognitive limitations (e.g., dementia, mental retardation, substance abuse and psychiatric disorders).

Episodes of Type II WPV seem to occur more frequently in emergency and psychiatric wards. This result is in line with the consideration of the Ministry of Health [69] and previous studies e.g., [58,59]. Nurses seem to be the category most involved. They are probably exposed to a greater risk of violence because they work in direct contact with patients and with their relatives, who are often emotionally vulnerable, frustrated and prone to loss of control [70]. However, in the literature, the interest in the phenomenon of violent behavior aimed at the nursing profession is relatively recent. In fact, in the 1980s and 1990s, attention was paid to other professions, such as psychiatrists, psychologists, etc. Only later did nurses become an interesting subject [25]. An explanation for this is probably to be found in the reference model for the management of care. We have gone from a model that dealt only with the treatment of the disease to a model that instead concerns itself with the prevention and promotion of well-being. From this perspective, the role of nurses and their function have therefore also changed. This has also brought about environmental and structural changes. Cuts have been made to control costs, and the average length of stay has decreased; the number of hospital beds has also decreased, and fewer nurses are employed due to staff reductions. However, at the same time, there has been an increase in the number of patients, perhaps also as a consequence of the factors mentioned above, which have decreased the quality of care. All of these changes have contributed to increased stress both for patients and nurses and therefore have heightened the risk of violence occurring. For this reason, research on nurses is likely to increase further [25]. With regard to the nurses’ work, on a global level, the following characteristics of patient and health care provider interactions contributing to WPV were found in other studies: misunderstandings or disputes regarding medical issues [71], patients thinking that they are not being taken seriously [71], dissatisfaction with treatment or physicians [72,73], physical contact during the provision of care or during a physical assessment [74,75] that is accompanied by pain or that crosses boundaries relating to intimate areas or private spheres [76], frustration with the patients’ intention, enforced personal care and enforced medical treatment [75] or the disagreement over care provision and treatment by staff [75] or by the patient [72,76]. Aggressive behaviors seem to be often generated by concern for health conditions, long waits, the perception of poor quality of care received and the resulting dissatisfaction with the attitudes of operators [77]. Young and inexperienced operators are more exposed to attacks, while greater seniority and even age per se seem to be protective factors [54].

Overall, there is a low propensity on the part of health workers to report the violence suffered [13,25,26,27,46,78]. There is a tendency to tolerate the WPV, to consider it an inevitable part of the job in hospitals and to justify it as not knowingly intended by the patients who commit it. Furthermore, the victims are often afraid of being potentially reproached and do not trust their organizations to handle the matter properly [79,80]. Findorff and colleagues [81] (p. 399) define the concept of “below reported” as situations in which “an individual is victimized and does not report the incident to an employer, the police or through other means”. In the Italian context, Papalia and Magnavita [82] speak of a real “specific occupational risk”. In part, an explanation of this phenomenon could be found in the fact that in the health sector acts of violence are perceived and accepted as phenomena belonging to the work itself, and for this reason, accidents are not reported [13,26,27,82,83,84,85,86]. In addition to this motivation, other explanations can be sought. First of all, we must consider that many workers think that reporting attacks or episodes that happened to them do not work in their favor and might even hinder them. In addition, the accident can also be perceived as an episode in which they were ineffective and not up to the task. Nurses may have the idea that reporting the incident and then admitting that it happened entails a form of negligence and lack of adequate skills [26,27,78]. Work-related stress also plays an important role: if a worker is under stress, the response to violent events could be magnified. Stress can favor the alteration of responses [66].

The under-representation of the Southern Italian regions as research locations is a further key point worth noticing. Indeed, richer areas, such as North and Center Italy, spend more than the South on hospital and residential care as well as on the overall healthcare system, investing less in prevention and worker protection policies [87]. Moreover, in the poorer areas, it occurs more frequently that health services are administered on a secondary care level, with a population that is usually far away from health structures and approaches them only in case of need [88], i.e., when the stress levels are already higher.

A further consideration is that there is not a common description of violence throughout the selected studies, nor a unique tool (questionnaire, interview, checklist) used to evaluate the phenomenon. The recent publication in the Official Gazette n.20 of 26 January 2021 of the Law n. 4 of 15 January 2021 on the elimination of violence and harassment in the workplace, could represent a clearer yardstick in defining what can be considered violence. A great difficulty in adopting intervention policies regarding violence in the workplace has in fact often been characterized by the opacity with which it is defined and by the arbitrariness of judgment by victims, perpetrators and institutions. Issues in recognizing healthcare violence and/or fear in reporting violent episodes are the most frequent causes of under-reporting [13,60,89]. In conclusion, the importance of specific training and of promoting a conscious culture and the need for laws and interventions to protect healthcare workers emerge forcefully.

The interest of the scientific and institutional world for the phenomenon of violence in the workplace is rapidly increasing. In order to implement an effective program, it is useful to set up monitoring tools and diverse and multi-disciplinary working groups. If we dwell on the various levels of analysis, some research affirms that between individual factors and corporate and socio-environmental factors, the ones that predict hostile acts the most are situational factors [25,31,90]. However, focusing on these factors alone or considering them individually might be insufficient. Cooper and Swanson [25] developed a conceptual model for understanding how the factors that cause violence in the workplace interact with each other. In the model, attention is focused on the interaction between workers and aggressors, who should not be considered as individual protagonists of the violent act, for the interconnectedness between actors represents an additional factor in its own right. Alongside this worker–aggressor relationship, three other levels of factors are involved: business, community and society. The peculiar aspect of this model is that the three levels of factors form concentric circles that revolve around the relationship between worker and aggressor. It is therefore important, when talking about violence, to use a model that is multi-factorial and that sees these factors as interconnected. Otherwise, the risk is to jump to the false conclusions that violence is caused either only by intra-personal factors or only by interpersonal and/or socio-environmental factors. It is therefore important to address the phenomenon of violence in the workplace with an integrated approach, since it is a complex phenomenon in which the whole is more than the sum of its parts [83]. The organizational characteristics contributing to WPV included procedures to check the patient’s identity [76], prolonged waiting times [72,73,76,91], unavailability of a doctor [75,76], anger about hospital policies and rules [76,91], the toxicity of the workplace environment, which indeed has an effect on employee’s engagement [92], and discharge procedures [83].

### Limitations

A first limitation, relating to the research method, concerns the possibility that some relevant studies may not have been included, as they were published in a language other than English or Italian or in journals not indexed in the databases in which the searches were conducted. Secondly, due to the great methodological variety of the selected studies (in some cases, the reported studies used ad hoc questionnaires), the non-homogeneous time of observation and the different geographic areas studied, it is difficult to exactly quantify the phenomenon in a definitive way. Another very important element concerns the subjectivity of HCW victims of violence, because it is important to note that the interpretation of the episodes of aggression are not perceived in the same way, but that they vary according to the personality of each subjects. Therefore, since all the studies selected in this paper refer to self-report tools, this situation may not be comparable between different subjects. Finally, both workers and public opinion could recognize violence in the workplace as an integral part of their work, considering it as something normal, underestimating and therefore not reporting the episodes of violence. Generally, the results presented here are a synthesis of the research literature and therefore share the limits of the original research, such as the risk of under-reporting, the voluntary nature of the interviewees, the lack of homogeneity in the definition of violence.

## 5. Conclusions

Based on the results of our scoping review, we can conclude that workplace violence is a phenomenon present among healthcare workers in Italy. Between 11.9 and 93.3% of HCWs reported having been victims of verbal aggressions and threats, while 27.5–50.3% claim to have been victims of physical violence. Considering the detection difficulties and the large methodological heterogeneity in monitoring the problem, it is recommended that the registers of aggression be standardized throughout the Italian territory and an adequate policy be structured in order to promote a greater awareness of HCW on the issues of violence in the workplace. More training would be needed, as well as carrying out targeted periodic dissemination campaigns, underling the importance of reporting all aggressions and providing specific information on the definition of the concept of “workplace violence”. In this way, it could be possible to obtain more information, to better monitor the phenomenon and implement more effective prevention and intervention protocols.

## Figures and Tables

**Table 1:** List of included studies, with research location and tool(s) used for the investigation.

First Author	Year of Publication	Research Location	Sample, Response Rate in %	Tools
Romito et al.	2004	Public hospital (Trieste)	265 healthcare workers (Physicians and nurses). Response rate = 61.6%	Sexual Experiences Questionnaire modified
Grottoli et al.	2007	-	355 healthcare workers	-
Camerino et al.	2008	General Hospital (outpatient departments, surgery, geriatrics, medicine, pediatrics and psychiatric departments)	5541 nurses. Response rate = 61.6%	Ad-hoc questionnaire
Zampieron et al.	2010	94 different clinical units (outpatient departments, surgery, geriatrics, medicine, pediatrics and psychiatric departments of two Italian health institution	595 nurses. Response rate = 85%	Ad-hoc questionnaire
Catanesi et al.	2010	-	1202 psychiatrists’ members of the Italian Society of Psychiatry. Response rate = 20.2%	Ad-hoc questionnaire
Cerri et al.	2010	-	467 healthcare workers	-
Magnavita & Heponiemi	2011	General Hospital	346 nursing students and 275 nurses from a general hospital. Response rate = 94.2%	Violent Incident Form
Magnavita et al.	2012	Public hospital	992 radiologists	Violent Incident Form
Magnavita & Heponiemi	2012	A general hospital and Public Health Care Facilities	1166 healthcare workers. Response rate = 80.1%	Violent Incident Form
Mastronardi et al.	2013	42 public mental health centers	478 psychiatrists: 246 publics and 232 privates.	Ad hoc interview
Gagliardi et al.	2013	-	396 professionals	-
Grattagliano et al.	2014	Public mental health centers and private psychiatrists (Bari)	101 healthcare workers (doctors, psychologists, nurses, socio-health workers).	Adhoc questionnaire
Magnavita	2014	Health unit (Rome)	698 healthcare workers. Response rate = 96.5%.	Violent Incident Form
Acquadro Maran et al.	2014	Different wards of 4 Italian state hospitals located in the North of the country	765 nurses from different wards (obstetrics, emergency internal medicine, otolaryngology and pediatrics). Response rate= 38.4%	The modified Italian version of the Questionnaire constructed by The Network for Surviving Stalking (NSS)
Terzoni et al.	2015	Different wards of a major Italian hospital	903 healthcare workers (336 nurses, 195 medical doctors, 109 administrative employees, 52 auxiliaries and 47 physiotherapists, 164 included laboratory technicians, workmen, midwives, professional educators, auxiliary personnel, biologists, head nurses, or did not specify). Response rate = 48.7%	ISTAT (Istituto nazionale di statistica) questionnaire
Luciani et al.	2016	A major Italian Hospital (Lombardy Region)	198 nurses	-
Ferri et al.	2016	15 wards of a general hospital	419 professionals (77 physicians, 17 head nurses, 259 nurses, 66 nursing assistants). Response rate = 56.2%	Violent Incident Form
Cannavò et al.	2017	Emergency Departments of a general hospital	51 healthcare workers (administrative staff = 4, physicians = 5) and nurses = 42). Response rate = 87.9%	Questionario sulla Violenza in Sanità
Acquadro Maran et al.	2018	A hospital in northern Italy	108 healthcare workers and 96 volunteers working in cardiology and oncology wards	Violent Incident Form
Ramacciati et al.	2019	All the Italian Emergency Departments	816 Emergency nurses in all Italian regions. Response rate = 7%	Questionario per l’Indagine Nazionale 2016 sulla Violenza verso gli Infermieri di Pronto Soccorso
Berlanda et al.	2019	Eight Emergency departments in northeastern Italy	149 (87 Physician and 62 nurses). Response rate = 37.7%)	Adhoc questionnaire
Cannavò et al.	2019	An Emergency Department of a general hospital and an acute psychiatric inpatients unit	323 healthcare workers (nurses, auxiliary and administrative staff). Response rate = 80.7%	Health Violence questionnaire
Franchini et al.	2020	Private Hospital (Milan). Rehabilitative psychiatric and neurological wards	55 healthcare workers (41 nurses, 6 healthcare assistant, 4 residents, 4 social educators).	A semi-structured interview
Ferri et al.	2020	An emergency department of a general hospital	27 Italian nurses involved in the triage area. Response rate = 100%	Violent Incident Form
Bizzarri et al.	2020	Psychiatric Services (Bolzano)	164 mental health workers. Response rate = 77.7%.	Risk Analysis Questionnaire
Magnavita et al.	2020	General Hospital	275 nurses. Response rate = 91.1%	Violent Incident Form
Viottini et al.	2020	University Hospital (Turin)	10,970 healthcare workers (nurses, medical doctors, support staff, administratives)	Aggression Reporting Form
Zoleo et al.	2020	Three emergency departments of a teaching hospital (Padua)	171 healthcare workers (Nurse, Physician and Patient care assistant) from general, pediatric and obstetric-gynecological emergency departments.	Ad-hoc questionnaire
Firenze et al.	2020	Doctors from Northern Italy	4545 healthcare workers.	Ad-hoc questionnaire
Aguglia et al.	2020	Emergency psychiatric wards in 3 Hospitals and 3 Mental Health Centres	183 mental health workers: nurses = 56, psychiatrists = 39, residents = 58, other professionals = 30. Response rate = 67%	Ad-hoc structured questionnaire
Gravante et al.	2020	Emergency Departments in Campania Region	83 emergency nurses	-
Converso et al.	2021	Two large hospitals in northern Italy	300 nurses. Response rate = 60%	Violent Incident Form

**Table 2:** Percentage of HCWs who have experienced WPV in the last 12 months.

Authors	Profession	Workplace Violence in %			Patients Violence in %		Visitors Violence in %	
		Verbal	Physical	Harassment	Verbal	Physical	Verbal	Physical
Zampieron et al. 2010	Nurses	81.6	4.8	-	-	-	-	-
Romito et al. 2004	Nurses and physicians			29	-	-	-	-
Magnavita et al. 2011	Physicians Nurses Psychiatrists others	11.9	9.2	23	-	-	-	-
Magnavita andHeponiemi, 2011	Nurses Nursing students	34.9	9.5	-	65.4		14.7	
Magnavita et al. 2012	radiologists	16.3	5.9	27.6				
Converso et al. 2021	Nurses	85.8	6.6	-	36.8		59.8	
Aguglia et al. 2020	Mental health workers: Psychiatrists, residents, nurses and others	41.5	2.7	-	-		-	
Magnavita et al. 2020	Nurses	19.6	9.8	-	-		-	
Bizzarri et al. 2020	mental health workers	90.9	44.5	-	-		-	
Ramacciati et al. 2018	Nurses	76.0		-	-		-	
Acquadro Maran et al. 2018	HCWs in oncology and cardiology	50.5	25.0	-	-		-	
Ferri et al. 2020	Nurses	92.3	0	-	30.8		61.5	
Terzoni et al. 2015	Nurses Physicians Laboratory technicians Physiotherapists Auxiliaries Administratives	40.2	11.5	-	75.0	58.0	23.1	27.6
Magnavita, 2014	Physicians Nurses Technicians	52.6	24.6	-	-		-	
Berlanda et al. 2019	Physicians and Nurses	93.3	53.0	-	95.30	52.35	85.91	20.81
Firenze et al. 2020	Physicians	51.5	39.4	-	23.0	78.0	58.0	59.0

**Table 3:** Percentage of HCWs who have experienced WPV at some point during their career.

Authors	Profession	WPV in %			Patients Violence in %		Visitors Violence in %	
		Verbal	Physical	Harassment	Verbal	Physical	Verbal	Physical
Magnavita & Heponiemi, 2012	Physicians Nurses Psychiatrists others	65.5	25.7	5.5	50.8		23.1	
Magnavita et al. 2012	radiologists	48.8	30.0	20.7	32.5	37.1	15.9	34.3
Catanesi et al. 2010	Psychiatrists	90.9	64.6	72.0	-		-	
Aguglia et al. 2020	Mental health workers: Psychiatrists, residents, nurses and others	89.6	50.3	-	-		-	
